# Atypical Histopathological Aspects of Common Types of Lung Cancer—Our Experience and Literature Review

**DOI:** 10.3390/medicina60010112

**Published:** 2024-01-07

**Authors:** Angela-Ștefania Marghescu, Diana Gabriela Leonte, Alexandru Daniel Radu, Elena Doina Măgheran, Adrian Vasilică Tudor, Cristina Teleagă, Mirela Țigău, Livia Georgescu, Mariana Costache

**Affiliations:** 1 Research Department, “Marius Nasta” Institute of Pneumophthisiology, 050159 Bucharest, Romania; alexandru.radu@marius-nasta.ro (A.D.R.); cristina.teleaga@marius-nasta.ro (C.T.); mirela.tigau@marius-nasta.ro (M.Ț.); livia.georgescu@marius-nasta.ro (L.G.); 2 Pathology Department, “Carol Davila” University of Medicine and Pharmacy, 020021 Bucharest, Romania; mariana.costache@umfcd.ro; 3 Pathology Department, “Marius Nasta” Institute of Pneumophthisiology, 050159 Bucharest, Romania; diana.leonte@personalgenetics.ro (D.G.L.); elena.magheran@marius-nasta.ro (E.D.M.); adrian.tudor@marius-nasta.ro (A.V.T.); 4 Pathology Department, University Emergency Hospital, 050098 Bucharest, Romania

**Keywords:** lung, carcinoma, acantholytic, pseudoangiosarcomatous, signet ring cell, clear cell, WHO classification

## Abstract

Lung cancer is among the most common oncological diseases regarding incidence and mortality, with most of these having epithelial origins. Pathological reporting of these tumors is conducted according to the 5th edition of the World Health Organisation (WHO) classification of thoracic tumours. This study aims to draw the pathologist’s attention to four rare, atypical microscopic aspects that some of the most common types of lung malignancies reveal upon standard evaluation (hematoxylin-eosin stain) that make histopathological diagnosis challenging: acantholytic, pseudoangiosarcomatous, signet ring cell, and clear cell features. Each of these aspects was exemplified by a case diagnosed in the pathology department of the “Marius Nasta” Institute. Furthermore, we analyzed the classification dynamics of different WHO editions and used PubMed to review articles written in English and published in the last eleven years on this subject. Pathologists should be familiar with these unusual aspects to avoid misdiagnoses and to ensure the correct classification of tumors, which is extremely important because these tumor phenotypes have been associated with specific molecular alterations and a worse clinical evolution. There is a need to clarify the histogenesis and associated genetic mutations, given the fact that the rarity of these tumor phenotypes makes their study difficult. Some authors consider these to be overlapping entities; however, we do not encourage this, as they may exhibit different prognoses and various molecular alterations with important therapeutic implications. The signet ring cell feature was associated with ALK rearrangement in lung adenocarcinoma; thus, these patients can benefit from tailored therapy with ALK-tyrosine kinase inhibitors (ALK-TKI). Recent studies associated clear cell morphology with FGFR3-TACC3 fusion, suggesting that patients with this diagnosis may be potentially eligible for FGFR inhibitors. We described, for the first time, the pseudoangiosarcomatous pattern in a case of lung adenocarcinoma; to our knowledge this aspect has only been described until now in the context of squamous cell carcinomas.

## 1. Introduction

Lung cancer is one of the most common malignancies in the world, with an estimated 2, 206, 771 new cases in 2020, according to the Global Cancer Observatory (GLOBOCAN). This pathology has the highest incidence among oncological diseases in men (1, 435, 943 new cases in 2020), and the third highest incidence in women (770, 828 new cases in 2020), after breast and colorectal cancer [1]. 

It was responsible for 1, 796, 144 deaths in 2020 (1, 188, 679 men and 607, 465 women), being the first cause of mortality by malignant neoplastic disease for men, and the second for women (after breast cancer) [1]. 

By far the most frequently diagnosed types of lung cancer (about 85%) are malignant epithelial tumors (carcinomas), classified as non-small cell (NSCLC) or more rarely (about 15%), as small cell (SCLC) carcinomas. Adenocarcinoma and squamous cell carcinomas have always represented the most common types of NSCLC, but their proportions have changed over time in favor of the former. Even if pathologists are well-trained in recognizing these tumors, their diagnosis can sometimes be extremely challenging, as they may exhibit rare or unusual histopathological aspects [2].

Pathological reporting of lung tumors must be conducted according to the World Health Organization (WHO) classification of thoracic tumours, which reached its 5th edition in 2021. This is essential for establishing the histopathological type, grading, and staging of cancer; evaluating the prognostic factor and choosing the predictive testing to implement the tailored oncological therapy [2]. 

As these are dynamic aspects and new scientific achievements are continuously acquired, the scientific board periodically updates the WHO classification of tumours [2].

## 2. Materials and Methods

Four cases of lung cancer were selected from the database of the Pathology Department of “Marius Nasta” Institute. The histopathological diagnosis of the cases was challenging because of their atypical microscopic aspect upon standard evaluation ( hematoxylin-eosin stain-HE), which raised difficulties regarding their origin. We defined four particular microscopic aspects of the tumors that made the diagnosis difficult and focused our study on the acantholytic, pseudoangiosarcomatous, signet ring cell, and clear cell features. Immunophenotyping (immunohistochemistry stains) was performed for each of the selected cases. 

All the included patients were adults (aged over 18 years old), diagnosed with lung cancer on resection specimens (three surgical resections and one necroptic), with signed informed consent. 

We excluded lung metastasis, benign or rare types of lung cancer (for example, sarcomas or lymphomas), tumors with uncertain malignant potential, in situ lesions, and cases that lacked an immunohistochemical evaluation or signed informed consent.

Standard processing of the surgical specimens begins with tissue fixation in 10% formalin, followed by dehydration in increasing concentrations of ethanol (from 70% to 100%), clearing (in toluene), and paraffin embedding. Thin sections are performed on the paraffin block to stain them using hematoxylin-eosin (HE), special stains such as periodic acid–Schiff (PAS) and Grocott staining, or those used for performing immunohistochemistry (Leica Bond Max automated immunohistochemistry stainer was used). The slides were deparaffinized by toluene and 96% ethanol, exposed to antigen retrieval, and then incubated overnight with primary antibodies. The following immunohistochemical markers were used: AE1/AE3, TTF1 (SPT24 clone), napsin A, CK7, CK5/6, p40, CD31, CD34, PAX8.

Furthermore, we searched the actual and previous editions of the WHO classification of lung tumours to evaluate the dynamics of the lung adenocarcinoma and squamous cell carcinoma classification. 

Afterward, we reviewed the scientific database publication using PubMed to assess all the English-written articles published in the last eleven years, between 2013 and the present.

Table 1 summarizes some of the rare or unusual microscopic aspects of lung adenocarcinoma and squamous cell carcinoma, which represent the most common types of lung cancer. The table includes the keywords used in our search, the total number of returned articles, and the number of relevant articles representing the basis of our study.

## 3. Results

All four selected cases were confirmed by immunostains as a primary lung adenocarcinoma or squamous cell carcinoma (in context).

### 3.1. Case No. 1

A 64-year-old ex-smoker (40 pack-years, smoking cessation seven years ago) male, urban medium, with professional exposure to respiratory noxes (for ten years), presented with cough, dyspnea, and physical asthenia. From his medical history, we noticed type II diabetes and chronic obstructive pulmonary disease (COPD), grade GOLD 4.

The computer tomography (CT) evaluation showed a solid nodule in the pulmonary inferior left lobe, right lung emphysema, and bilateral bronchiectasis. The bronchoscopy revealed no abnormalities in the explorable territory.

A macroscopic examination of the surgical specimen revealed a white 3 cm left centro-hilar solid nodule, with black anthracosis pigment deposits and a firm consistency.

**Figure 1:**
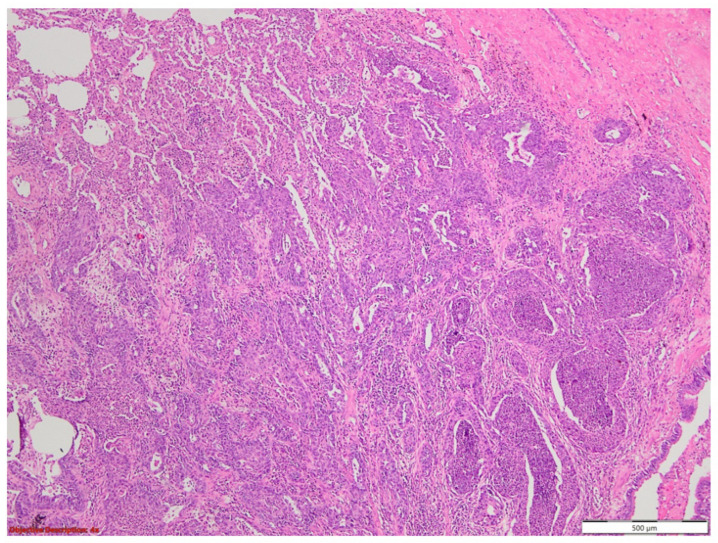
Lung tissue with tumoral proliferation formed by polygonal cells arranged in nests, with acantholytic aspects; HE, 40×.

**Figure 2:**
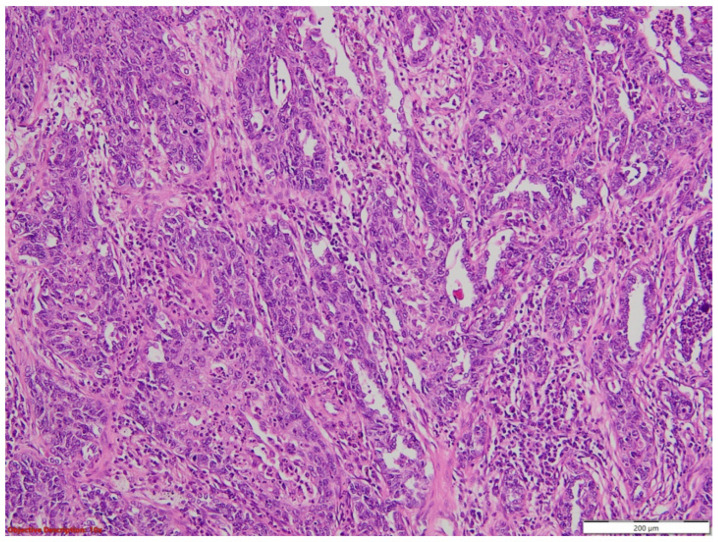
Squamoid carcinomatous proliferation with impaired intercellular junctions, creating pseudoglandular structures; HE, 100×.

The microscopic evaluation showed a tumoral proliferation formed by large, polygonal carcinomatous cells with vesicular nuclei and abundant eosinophilic cytoplasm; we noticed the loss of intercellular adhesion in the centrum of the tumoral nests, forming pseudoglandular structures (Figure 1 and Figure 2).

On the immunostains, tumoral cells were positive for p40 and CK5/6 and negative for TTF1 and napsin A, sustaining the diagnosis of lung squamous cell carcinoma with acantholytic features (in context).

### 3.2. Case No. 2

A 52-year-old smoking (32 pack-years) female from an urban medium, with no professional exposure to respiratory noxes, presented with hemoptysis, cough, inappetence, and physical asthenia. The patient mentioned an atopic background, without any other significant medical history. 

The CT evaluation revealed a consolidation mass, with central necrosis in the upper right lobe of the lung, emphysema, and bilateral hilar adenopathy. The bronchoscopy revealed an inflammatory aspect, without any proliferative elements in the explorable territory, an aspect sustained also by the microscopic examination of the bronchoalveolar lavage.

**Figure 3:**
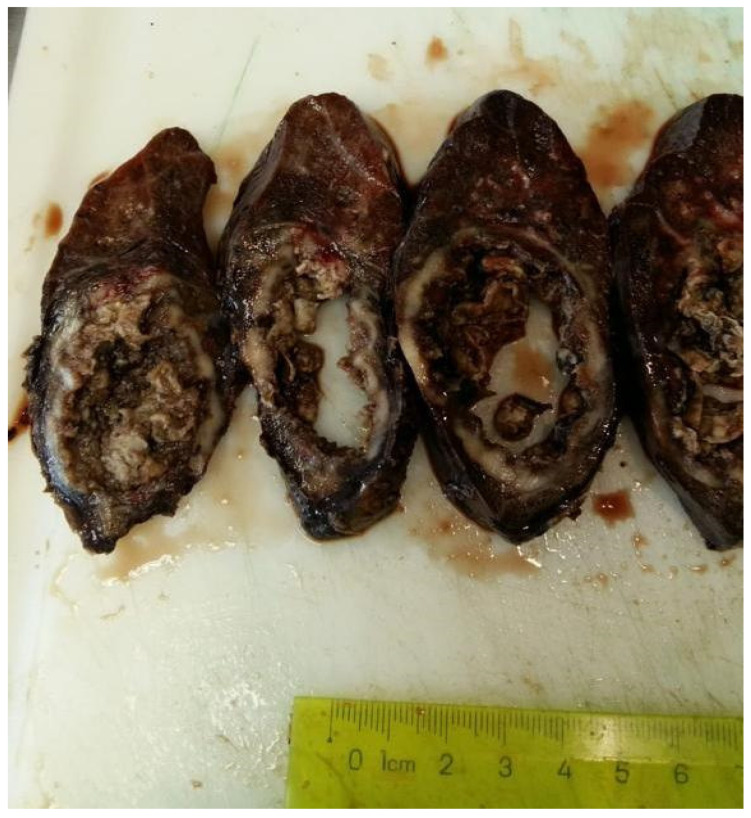
Gross examination of the surgical specimen showed a 55 mm grey tumor, mostly necrotic and abscessed.

Clinical and paraclinical tests raised the suspicion of tuberculosis. Even if the microscopic examination of the sputum and GeneXpert did not detect Koch bacillus, because of the high clinical and imagistic suspicion, the patient received three months of anti-tuberculosis medication, without ameliorating the symptomatology. An upper right lobectomy was performed, and the gross examination revealed a 55 mm grey tumor, firm, mostly necrotic and abscessed (Figure 3).

**Figure 4:**
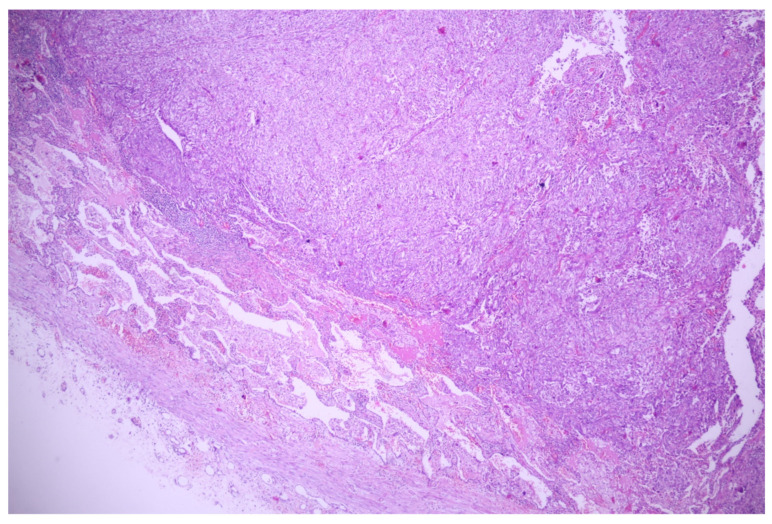
Undifferentiated lung tumor with a solid pattern of growth, HE, 40×.

**Figure 5:**
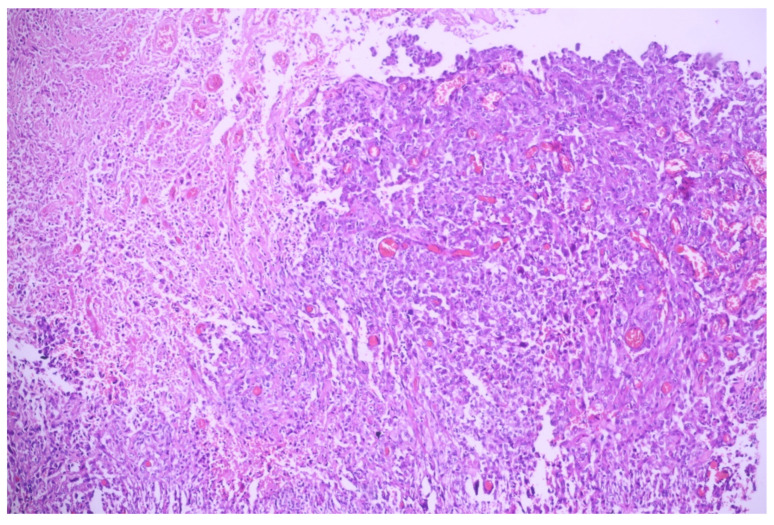
Diffuse tumoral proliferation with pseudoangiosarcomatous features and necrosis; HE, 100×.

Microscopic examination of the tumor revealed a malignant proliferation composed of large, atypical cells with pseudoangiomatous patterns of growth, forming pseudo-vascular tubular structures or slit-like spaces, which usually raise the suspicion of angiosarcoma (Figure 4 and Figure 5).

**Figure 6:**
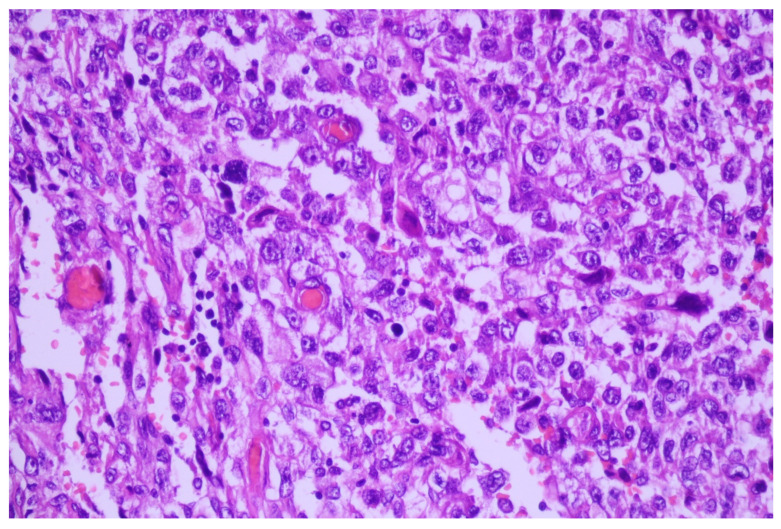
Malignant neoplasia formed by moderate pleomorphic cells, some of them appearing to line small blood vessels, others with evident emperipolesis; HE, 400×.

**Figure 7:**
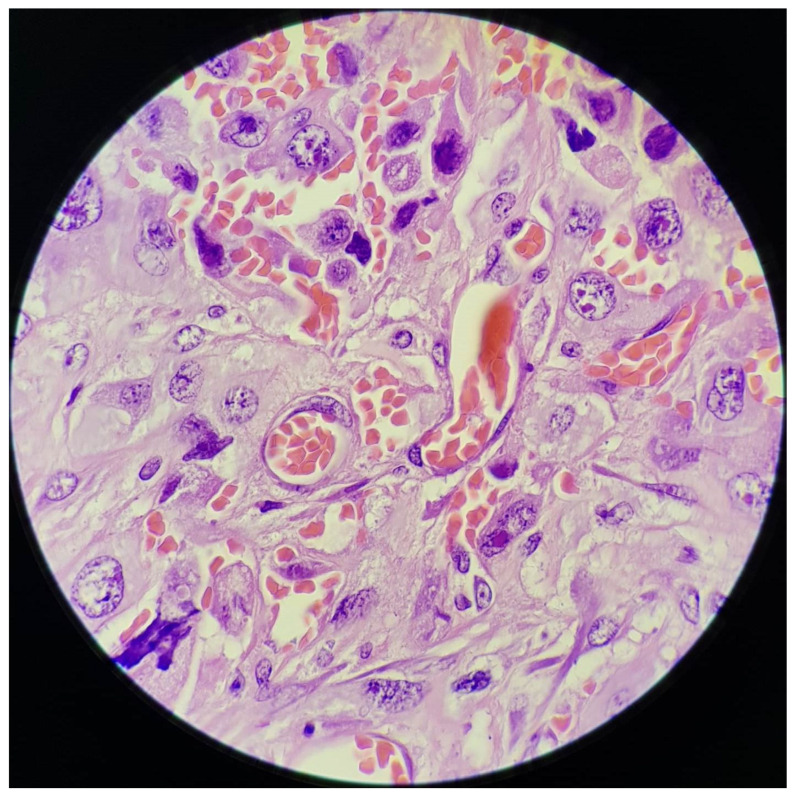
Emperipolesis—red blood cells found in the cytoplasm of the neoplastic cells; HE, 1000×.

The presence of emperipolesis (red blood cells found in the cytoplasm of the neoplastic cells) increased our suspicions even more (Figure 6 and Figure 7). 

**Figure 8:**
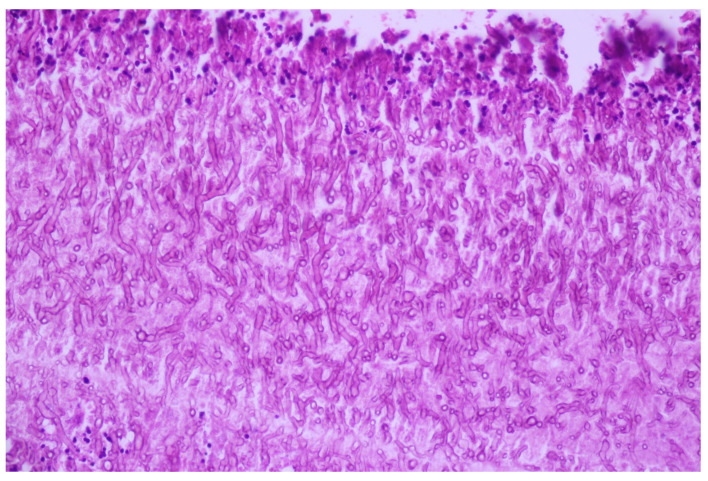
Septate hyphae of Aspergillus spp, with dichotomous or acute angle (<45°) branching; HE, 400×.

**Figure 9:**
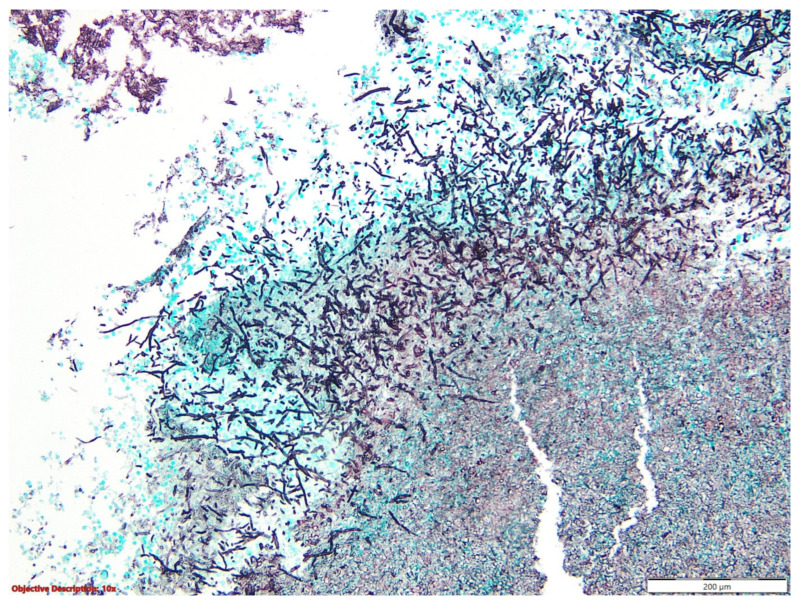
Aspergillus spp. hyphae are stained in black, on a blue background; Grocott-Gomori methenamine silver (GMS), 100×.

The central necrotic tumor area was colonized by fungus hyphae with dichotomous branching at 45-degree angles and fruiting bodies of Aspergillus spp. (Figure 8), positive at periodic acid–Schiff (PAS) and Grocott–Gomori methenamine silver stains (Figure 9). 

**Figure 10:**
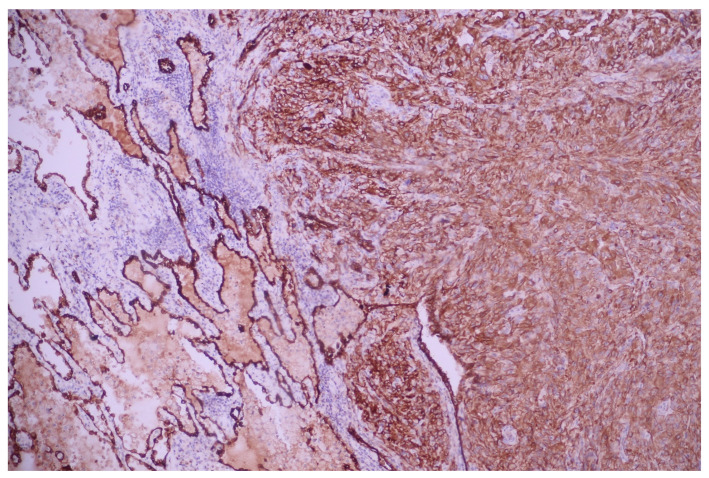
Pancytokeratin (AE1/AE3) positivity within the neoplastic cells; AE1/AE3 100×.

**Figure 11:**
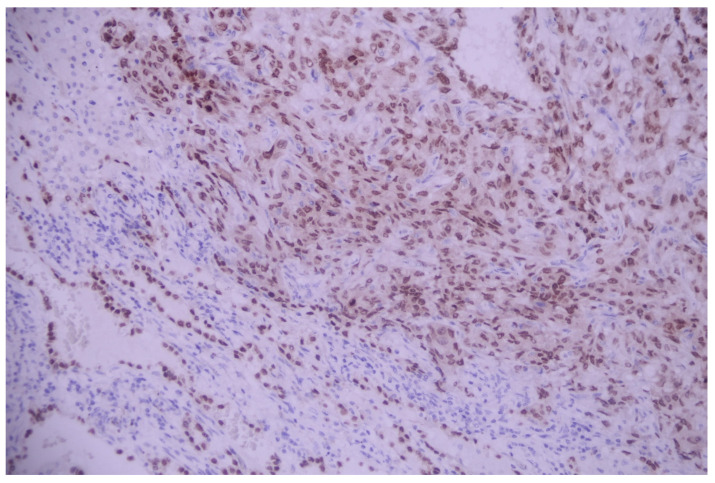
TTF1 nuclear expression within the malignant cells; TTF1, 200×.

**Figure 12:**
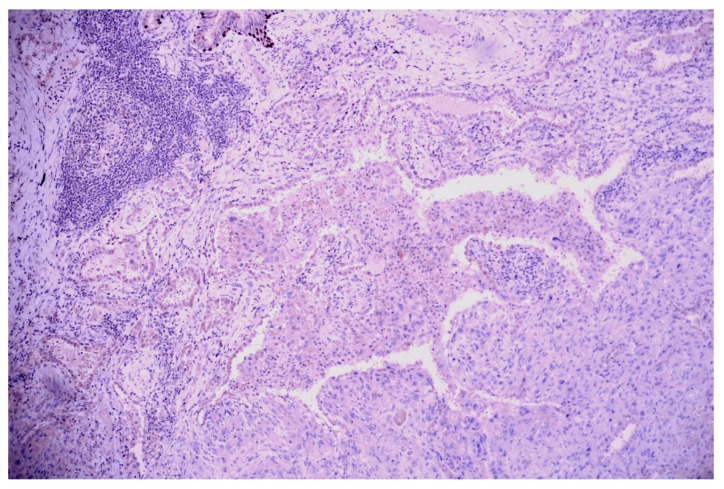
Tumoral elements are negative to p40 (internal control present in the bronchial basal cells); p40, 100×.

**Figure 13:**
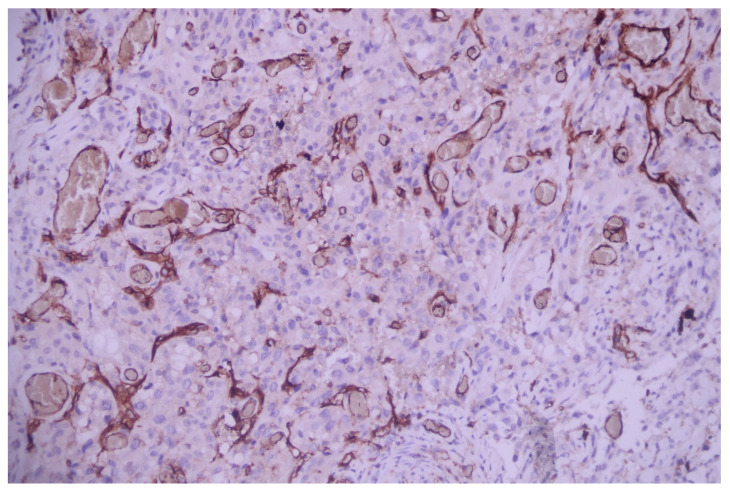
Malignant cells are negative to vascular marker CD31 (internal control present in the blood vessels); CD31, 200×.

**Figure 14:**
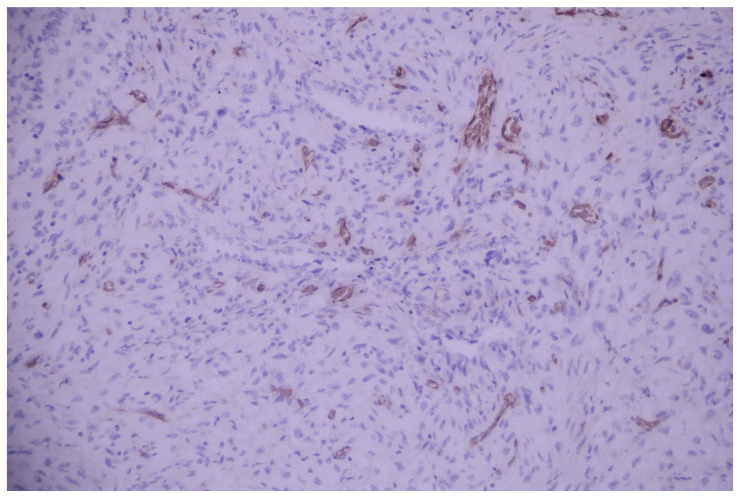
Malignant cells are negative to vascular marker CD34 (internal control present in the blood vessels); CD34, 200×.

Immunostains showed the positivity of neoplastic cells for pancytokeratin—AE1/AE3 and TTF1 and negativity for p40, CD31, and CD34 (Figure 10, Figure 11, Figure 12, Figure 13 and Figure 14).

The pathological diagnosis was lung adenocarcinoma with pseudoangiosarcomatous features, associated with Aspergillus spp. infection.

### 3.3. Case No. 3

An 88-year-old never-smoker female, urban medium, with no professional exposure to respiratory noxes, presented to “Marius Nasta” Institute with a dry cough, dyspnea, and profuse sweating. 

The patient was known to have grade III arterial hypertension with high additional risk, therapeutically neglected type II diabetes (blood glucose level: 239 g/dL at admission), a mild form of SARS-CoV-2 infection (in 2022), and Alzheimer’s disease. 

The CT evaluation showed a 40 mm solid mass in the upper left lobe of the lung, with multiple satellites, smaller nodules, left pleural effusion, ‘tree-in-bud’ interstitial appearance in the medium lobe, and fibrosis with consequently traction bronchiectasis in the right inferior lobe.

Because the patient’s general medical condition deteriorated rapidly, bronchoscopic evaluation and pleural punction were not possible.

The patient died four days after admission, and a necroptic examination was performed, which confirmed the malignant nature of the pulmonary solid mass, as well as the presence of multiple metastases (localized in the left upper and lower lobes of the lung, left visceral and parietal pleura, retrosternal and paravertebral in the thorax). Associated findings included: pulmonary thromboembolism, interstitial fibrotic changes of the lung parenchyma (most probably secondary, in the context of SARS-CoV-2 infection), atherosclerosis, simple left kidney cyst, and plurivisceral hyperemia.

**Figure 15:**
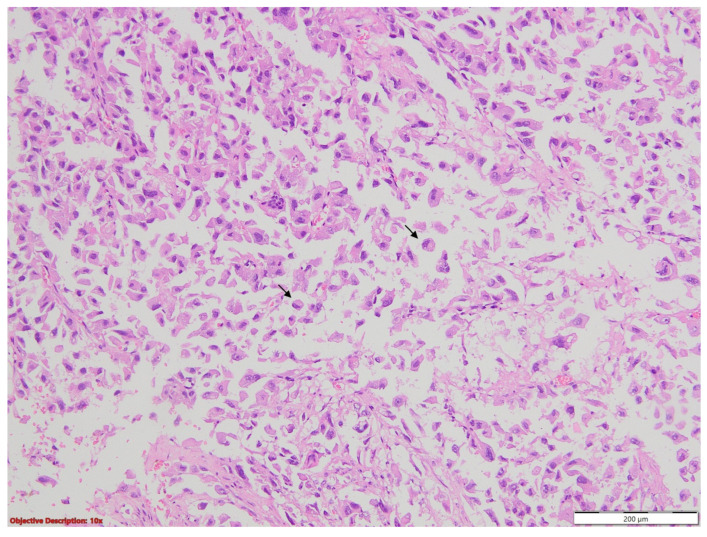
Carcinomatous proliferation with signet ring features (→); HE, 100×.

**Figure 16:**
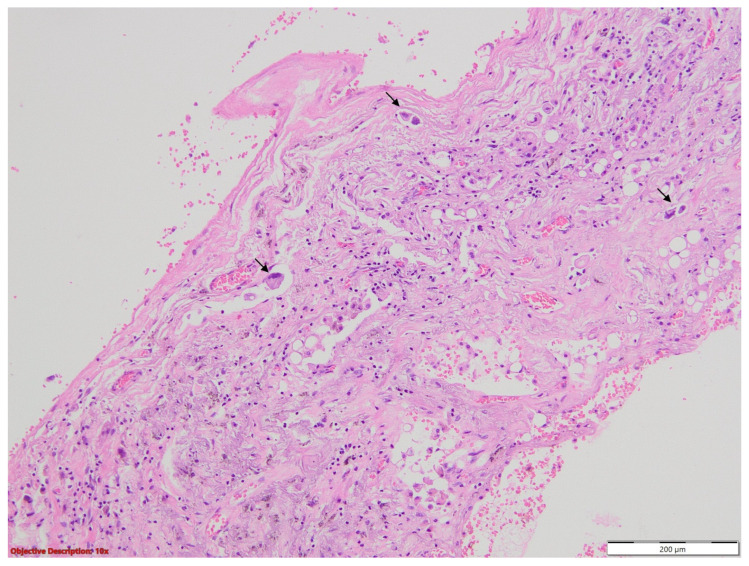
Lymphovascular invasion—medium-sized malignant cells (→) with peripheric nuclei (signet ring morphology) invasive in the lymphatic vessels of the pleura; HE, 100×.

**Figure 17:**
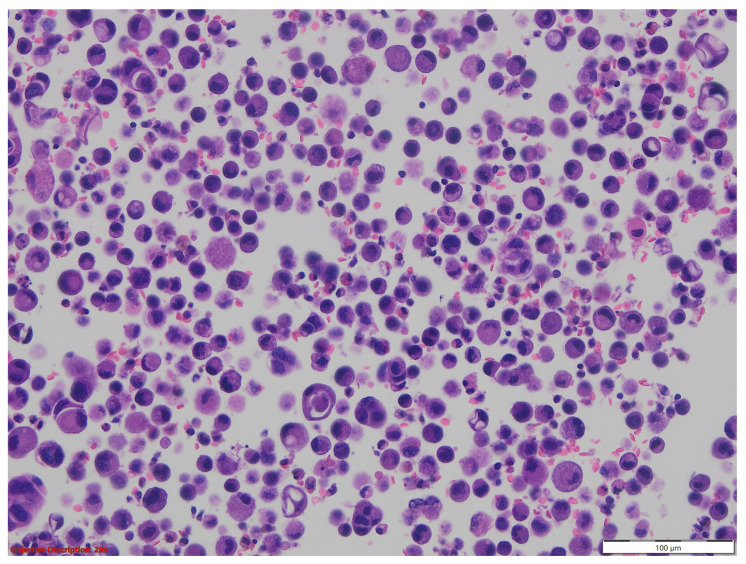
Pleural effusion (cytoblock)—tumoral cells having eccentrically nucleus and eosinophilic cytoplasm (‘signet ring’ cells), scant inflammatory and red blood cells; 200×.

Lung tissue fragments collected for the microscopical examination revealed carcinomatous neoplasia with lymphovascular invasion, having predominantly solid growth, focal micropapillary, and about a 15% atypical signet ring cell component (Figure 15 and Figure 16); we also performed a cytoblock from the pleural effusion, in which the same malignant cellularity was present (Figure 17).

**Figure 18:**
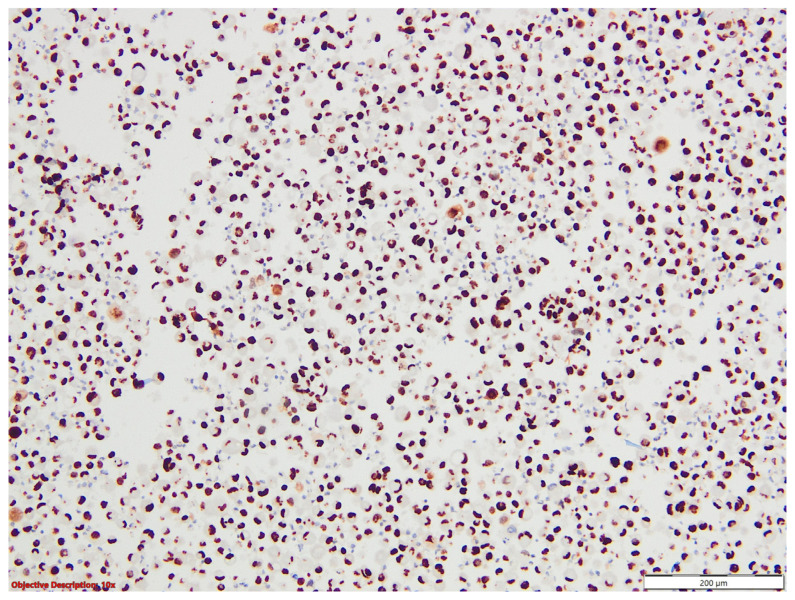
Pleural effusion (cytoblock)—a diffuse and intense nuclear expression of TTF1 within the neoplastic cells; TTF1, 100×.

**Figure 19:**
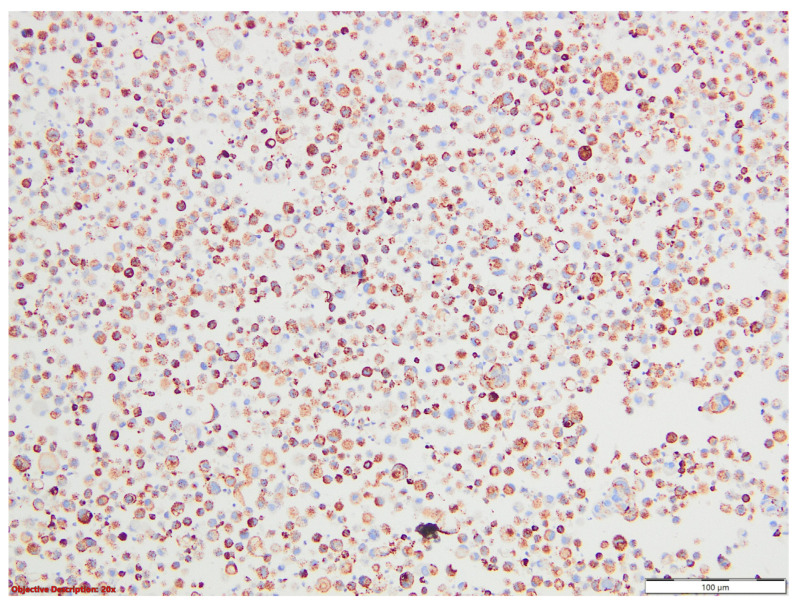
Pleural effusion (cytoblock)—a diffuse and intense cytoplasmic expression of napsin A within the neoplastic cells; Napsin A, 200×.

On immunostains, tumoral cell positivity for TTF1 and napsin A suggested a malignity with a pulmonary phenotype, compatible with the diagnosis of adenocarcinoma with signet ring features (Figure 18 and Figure 19). 

### 3.4. Case No. 4

A 69-year-old smoking (40 pack-years) man from an urban medium, with no professional exposure to respiratory noxes, presented to “Marius Nasta” Institute with medium hemoptysis, thoracalgia, and weight loss (8 kg in the last three months). From the medical history of the patient, we noticed chronic obstructive pulmonary disease (COPD) grade GOLD 2, lung tuberculosis (in 2015), severe obstructive sleep apnoea (OSA), atherosclerosis, arteriopathy obliterating the lower extremities (PAD), and bilateral glaucoma.

Computer tomography revealed a 30 mm left centro-hilar mass, bilateral bronchiectasis, mediastinal adenopathy, and left nodular hypertrophy of the suprarenal gland (one nodule of 17 mm). Bronchoscopic examination showed an endobronchial tumor in the left subsegmental bronchia, which was biopsied. The bronchial aspirate was positive for Klebsiella pneumoniae and negative for Koch bacillus. 

Macroscopic examination showed a 31 mm white and firm tumor, ill-defined borders, and necrosis; the tumor has a centro-hilar localization, with extension in the left lower lobe. 

**Figure 20:**
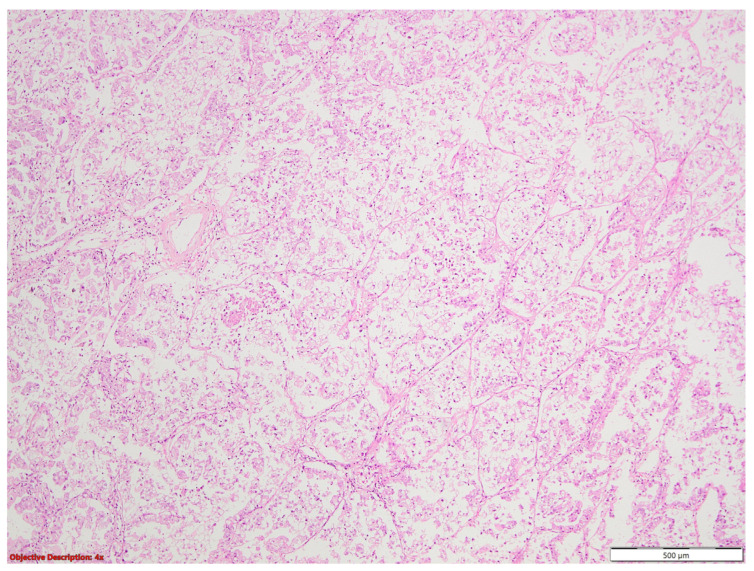
Carcinomatous proliferation with clear cell features; HE, 40×.

**Figure 21:**
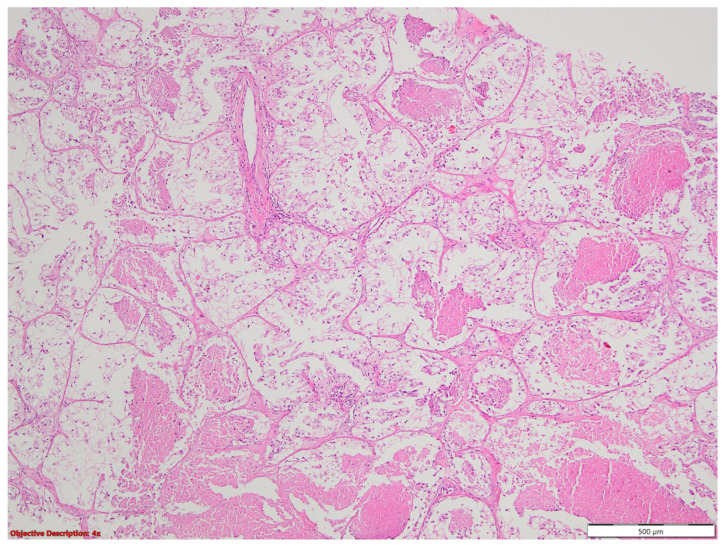
Malignant neoplasia formed by tall or polygonal cells with an abundant clear cytoplasm and atypical, hyperchromatic nuclei and tumoral necrosis; HE, 40×.

Microscopic examination revealed a malignant epithelial proliferation with a polymorph pattern of growth (predominant micropapillary and acinar, and focal solid, papillary, and lepidic). The tumor has an approximately 35% clear cytoplasm cell atypical contingent (Figure 20 and Figure 21). Space through the airspace spread (STAS) and lymphovascular invasion were present. 

The immunostains show positivity to TTF1, napsin A, and CK7 and negativity to PAX8 and CD10 of the neoplastic cells, sustaining a pulmonary phenotype; therefore, we signed the report as primary lung adenocarcinoma with clear cell features.

## 4. Discussion

### 4.1. WHO Classification of Lung Adenocarcinoma and Squamous Cell Carcinoma

#### 4.1.1. Lung Adenocarcinoma

According to the latest edition (5th edition) of the WHO classification of thoracic tumours, lung adenocarcinomas can have five different patterns of growth (lepidic, acinar, papillary, solid, and micropapillary). Regardless of the architecture, an evaluation of the cytological features is very important, helping us to classify the adenocarcinoma as non-mucinous, or less often as mucinous or mixed (if more than 10% of each component is present in one tumor). Mucinous adenocarcinomas have small, basal nuclei, usually with minimal atypia and a well-represented cytoplasm, containing mucin at the apical side of the cell (“goblet and/or columnar cell morphology”) [2].

Particular types of lung adenocarcinomas with low incidences, recognized by the 5th edition of the WHO classification, are colloid, fetal, and enteric types [2]. 

The WHO classification indicates that, regardless of the architecture, lung adenocarcinoma may have contingents of clear cells or signet ring cells. At present, these are not recognized as specific histopathological forms of lung adenocarcinoma, but pathologists are encouraged to mention in their reports the percentage of these cytological aspects because this could be important for the evaluation of tumor recurrences or metastasis, as well as negative prognostic features [2].

Over time, lung cancer classifications in the WHO editions have been dynamic. We searched in the previous editions of the last two WHO classifications to evaluate how the classification of lung tumors has changed [2,3,4].

The 3^rd^ edition of the WHO classification, published in 2004, stated that approximately 80% of resected lung adenocarcinomas were mixed, signifying that many different histologic subtypes could be objectivated in the same tumor, including acinar, papillary, solid (with mucin production) and bronchioloalveolar subtypes. Otherwise, the adenocarcinoma could be composed of a single pattern and classified in consequence [3]. Mucinous, nonmucinous, or mixt (mucinous and nonmucinous) tumors were also recognized, as in the 5th edition, with the difference being that the 10% cut-off needed for each component was not established [2,3]. 

Particular types of adenocarcinomas mentioned in the 3rd edition of the WHO classification included fetal, mucinous (also known as colloid), mucinous cystadenocarcinoma, signet ring cell, or clear cell adenocarcinomas. In the last two types of adenocarcinoma, the specific cytological features were usually focal; the blue book does not mention the percentage necessary for a tumor to be classified as this [3]. 

In the 4th edition of the WHO classification, published in 2015, important changes in lung tumor classification were made compared to the previous 3rd edition (2004). We tried to summarize the most important of these in the following paragraphs [4].

1. Nomenclature change: bronchioloalveolar adenocarcinoma (mucinous/non-mucinous, invasive/minimally invasive/in situ) became lepidic adenocarcinoma (mucinous/non-mucinous, invasive/minimally invasive/in situ) [4];
2. Micropapillary adenocarcinoma became a separate histopathological type, in addition to lepidic, acinar, papillary, and solid. The presence of micropapillary structures was also described in the 3rd edition of the WHO classification, but were included in the papillary histotype of lung adenocarcinoma [4];
3. Differentiation between mucinous and colloid lung adenocarcinoma: both neoplasms are formed by cells with the same morphology, the difference being that in the colloid tumors, the extracellular mucin deposits are extensive, fulfill, and break the alveolar septa [4];
4. Increase in the diagnostic accuracy of mixed mucinous and nonmucinous adenocarcinoma: introduction of a 10% cut-off as a minimum percentage needed for each component [4];
5. Elimination of some of the particular patterns of adenocarcinoma, including mucinous cystadenocarcinoma, signet ring, and clear cell adenocarcinoma [4];
6. Introduction of a new particular pattern of adenocarcinoma: enteric type [4].

The 5th edition of the WHO classification maintained the same classification of lung adenocarcinomas, the novelty being the presence of a more accurate proposed grading system for nonmucinous adenocarcinomas [2].

#### 4.1.2. Lung Squamous Cell Carcinoma

According to the last edition (5th edition) of the WHO classification of thoracic tumours, there are three recognized histopathological types of lung squamous cell carcinoma: keratinizing, non-keratinizing, and basaloid [2]. 

Clear cell or spindle cell, papillary, and alveolar-filling patterns are mentioned in the WHO classification as potential histological aspects of squamous cell carcinoma, without representing a different histopathological type of cancer (even if a significant part of the tumor has these features) [2].

The 3rd edition of the WHO classification recognized six different histopathological types of lung squamous cell carcinoma: keratinizing, non-keratinizing, papillary, clear cell, small cell, and basaloid. The clear cell variant was defined as a tumor formed “predominantly or almost entirely of cells with clear cytoplasm”, without mentioning a percentage of cells with this morphology mandatory for the classification of such a tumor [3]. 

The 4th edition of the WHO classification excluded three of the six squamous cell carcinoma histopathological types: papillary, clear cell, and small cell variants because the studies performed between 2004 and 2015 did not prove the molecular or prognostic significance of these microscopic aspects [3,4].

The 5th edition of the WHO classification maintained the same classification of lung squamous cell carcinomas as the previous one. As opposed to adenocarcinoma, the grading of squamous cell carcinoma is not postulated in the blue book; the interpretation is very subjective and difficult to reproduce in order to have a common language for all the medical specialists involved in the diagnosis and treatment of this pathology [2].

The WHO classification defines pleomorphic carcinoma (a type of sarcomatoid carcinoma) as a tumor represented by adenocarcinoma or squamous cell carcinomas with a contingent of ≥10% of giant and/or spindle cells [2].

WHO specialists continuously reevaluate the microscopical presentation of the neoplasms, and their molecular and epidemiological particularities, so the histopathological classification of the tumors undergoes periodic modifications to obtain an accurate diagnosis. The actualizations may consist of the introduction of new histopathological types of cancer, elimination, or the reclassification of a previously existing type [2,3,4]. 

### 4.2. Atipycal Histopathological Aspects of Common Types of Lung Cancer in Literature Review

After evaluating the dynamics of the pathological classification of lung cancer according to the WHO, we reviewed scientific database publications using PubMed to assess cases published in the last eleven years between 2013 and the present. 

#### 4.2.1. Acantholitic Features in Lung Carcinoma

The acantholytic aspect of a tumor is determined by the breakdown of the intercellular connections, with loss of cellular coherence, an aspect that may mimic glandular or vascular structures. This phenotype usually raises diagnostic difficulties, as the pathologist should rule out an adenocarcinoma or a vascular tumor, the latter especially when red blood cells are insinuating themselves between the tumoral cells [2,5,6].

There are few reported cases in the literature; all of these are presented in the context of squamous cell carcinoma. Most of these tumors were described in the skin, supporting the fact that the 5th edition of the WHO classification of skin tumours postulates acantholytic squamous cell carcinoma as a distinct histotype, opposite to the lung. The acantholytic form was never introduced in the thoracic WHO classification of tumors as a specific histotype [2,5,6,7,8,9,10]. Squamous cell carcinoma is typically formed by large, atypical, polygonal cells with abundant, eosinophilic cytoplasm, evident intercellular connections, and/or keratinization. The presence of acantholytic aspects determines the formation of pseudoglandular structures, mimicking an adenocarcinoma or adenosquamous carcinoma.

A study performed by Kenji Yorita et al. stated that, after the histological evaluation of skin squamous cell carcinoma with acantholysis, specialists divide this cancer into acantholytic (adenoid or pseudoglandular) and pseudovascular (pseudoangiosarcomatous or pseudoangiomatous) forms, both consisting of nests of tumoral cells with loss of the intercellular bridges in the central part of the nest, in contrast with the periphery, which is formed by cohesive cells [6]. Some specialists consider these forms as overlapping entities, but others support that they may have different pathways of metastasis; thus, they may require different clinical conduits. The pseudoangiosarcomatous form is even rarer than the pseudoglandular one; it was not included as a specific form of squamous cell carcinoma. Some authors consider these forms to be superposed, as the WHO classification of skin tumors states [3,5,6,9,11,12,13].

Signet ring squamous cell carcinoma is also presented in some articles as an acantholytic form of this cancer [7,8,10].

Even if it is known that tumors with an acantholytic morphology have a more aggressive clinical evolution and outcome compared with conventional forms, this was not postulated as a specific histopathological type of lung carcinoma in the actual classification, nor was it presented as such in the previous edition [2,3,4]. 

Kenji Yorita et al. sustain the hypothesis of epithelial-mesenchymal transition and a bidirectional evolution of the squamous cell carcinoma, between conventional and acantholytic forms, depending on the tumoral microenvironment. In the acantholytic zones, tumoral cells gain mesenchymal phenotype and lose E-cadherin, a fact that determines an increase in their motility and consequently a high risk of metastasis [6]. 

#### 4.2.2. Pseudoangiosarcomatous Feature in Lung Carcinoma

The pseudoangisarcomatous aspect of lung carcinoma supposes the presence of tumoral cells that line vascular-like anastomosing spaces which contain erythrocytes and, sometimes, floating neoplastic cells [11,12,13]. Red blood cells can sometimes be observed even in the cytoplasm of tumoral cells (as was seen in our case). These aspects are typically observed in the context of angiosarcoma, which should be excluded.

This was never recognized as a specific histological type of lung carcinoma, most probably due to its low incidence and prevalence [2,3,4].

In our research on Pubmed using the criteria mentioned previously, we found two articles published in the last eleven years (2013-present), of which only one was relevant. This was written by Yue Lin and al. and presented the case of a 68-year-old man with pseudoangiosarcomatous lung carcinoma with squamous differentiation, who was admitted to the hospital with hemoptysis, accentuated after the administration of antibiotics. CT examination revealed abnormalities (consolidation, ground-glass opacity) localized in the right lung [13]. 

Given the extremely low number of articles regarding pseudoangiomatous carcinoma published, we extended the research and found four articles, written between 1992 and 2023, with only three of them being relevant.

The first pseudoangiosarcomatous squamous cell carcinoma was mentioned in 1992 and, until now, only nine cases have been reported. Apart from the cases reported in 2021 by Yue Lin and al., we found eight cases of pseudosarcomatous squamous cell carcinoma reported in the lung, according to M. Kong et al. The gender ratio was M:F = 8:1 and the median age at diagnosis was 62.55 years old [11,12,13]. 

The medical literature reports that pseudoangiosarcomatous carcinomas usually affect patients of middle-age or older, who are long-term smokers, and who present at the hospital complaining of hemoptysis, cough, pain, or weight loss. Vasculitis and lung infections (fungal, tuberculosis) usually need to be ruled out [13].

In the 3^rd^ edition of the WHO classification, these tumors were classified under the large umbrella of pleomorphic carcinomas (a type of sarcomatoid carcinoma), describing the pseudoangisarcomatous aspect only in the context of squamous cell carcinoma. Its reserved prognosis was constantly observed [3]. In the 4th edition, this aspect was no longer mentioned [4].

To our knowledge, until now. the pseudoargiosarcomatous pattern has not been reported in the context of adenocarcinoma (regardless of its location).

#### 4.2.3. Signet Ring Features in Lung Carcinoma

Observing this cytologic feature in an otherwise poorly differentiated tumor favors, but is not specific to, the diagnosis of adenocarcinoma [14]. Lung adenocarcinoma is polymorph, with acinar, lepidic, micropapillary, papillary, and solid patterns of growth. The tumoral cells could vary from minimal to severe atypia, and mucin droplets are sometimes observed intracytoplasmic. Rarely, this may displace eccentrically the nucleus, determining a signet ring cell morphology. This aspect is strongly associated with adenocarcinoma, being most frequently encountered in gastric malignancies. The presence of these aspects in a lung tumor raises, first of all, the suspicion of metastatic gastric cancer, which should be ruled out by performing immunostains. The same applies to a clear cell morphology, which is frequently noticed in the context of clear cell kidney carcinomas.

The signet ring tumor cell has optically clear large vacuoles in the cytoplasm, which displace the nucleus to the periphery (in rare cases, the cytoplasm could appear as eosinophilic). The vacuole is the morphological expression of the presence of mucin, lipid, glycogen, or even immunoglobulin, or it may represent dystrophic changes (vacuolar degeneration) or artifacts; the latter is suspected when the vacuolar aspect is present both in the tumor and in the surrounding normal tissue [8]. Catalin Bogdan Satala et al. stated in their study that an electronic microscope examination of tumoral fragments with these features, collected from the skin, showed a rough endoplasmic reticulum with expanded cisternae [14].

The presence of signet ring neoplastic cells is most frequently reported in gastric carcinoma but can be present in malignant epithelial tumors with other origins, such as colonic, pancreatic, urothelial, breast, or pulmonary tumors [14].

The signet ring cell component was rarely reported in squamous cell carcinoma, with only 24 cases reported according to Catalin Bogdan Satala et al, most of these (14) in the skin [14]. Only three cases were found in the lung (one case reported in 2016 by Park, H. et al, one case reported in 2018 by Nuri Yiğit et al., and one case reported in 2019 by Handra-Luca et al.) [8,10,15]. The first case of squamous cell carcinoma with a signet ring cell component was described in 1988, in the skin, by Cramer and Heggeness [16]. In the lung, the first case was described 28 years later, by Park, H. [10].

Signet ring cells have been observed, extremely rare, even in non-epithelial malignant tumors, such as mesothelioma, lymphoma, gastrointestinal stromal tumor, or different types of sarcomas (angiosarcoma, liposarcoma or leiomyosarcoma) [14]. 

A study published by Catalin Bogdan Satala et al. confirms that the presence of the signet ring cells represents a step in the epithelial-mesenchymal transition in tumoral processes. This aspect is sustained by immunohistochemical tests, which show a different expression of the markers in the two tumoral contingents (signet ring and non-signet ring). The conventional squamous cell carcinoma has an epithelial phenotype, being positive to E-cadherin and beta-catenin, opposite to the signet ring component, which has a mesenchymal phenotype (diffuse expression of vimentin, beta-catenin with nuclear translocation and partially loss of E-cadherin). They also tested a marker of stem cells, of the sex-determining region Y-box 2 (SOX2), which has an important role in the survival and growth of the tumoral cells, being related to the progression and aggressive course of the tumors [14].

Regarding lung malignancy, the signet ring histological type was recognized only in the context of adenocarcinomas, in the 3^rd^ edition of the WHO classification [3]. Starting with the 4^th^ edition of the WHO classification, this histotype was eliminated [2,4].

It is very important to further analyze the implications of this phenotype’s presence in a tumor, given the prognosis and the potential therapeutical implications. Specialists state that lung adenocarcinoma with a signet ring morphology is often ALK-mutated, meaning that patients can benefit from ALK inhibitors [17,18,19,20,21,22].

#### 4.2.4. Clear Cell Features in Lung Carcinoma

Clear cell carcinoma has a clear cytoplasm, an aspect generated by the presence of glycogen, and a centrally localized nucleus (opposite to the signet ring cell) [5,14]. 

This was recognized as a specific histological type of adenocarcinoma and squamous cell carcinoma in the 3rd edition of the WHO classification, but were both eliminated from the classification starting with the 4th edition [2,3,4].

This represents a specific histopathological type of skin squamous cell carcinoma, the same as the acantholytic form. Even if it lacks a consensus, the WHO’s recommendation (5th edition) is that more than 25% of the tumor should have these features to classify as a neoplasia. The prognosis seems to be comparable to the conventional form of squamous cell carcinoma [5]. It can sometimes be very difficult to distinguish between signet ring cell, clear cell, acantholytic, and pseudoangiosarcomatous morphologies [14]. 

A recent study by Suster, D. et al. described, in a series of seven lung carcinomas with a clear cell phenotype, the presence of FGFR3-TACC3 gene rearrangement. The fusion of the fibroblast growth factor receptor 3 (FGFR3) to the transforming acidic coiled-coil 3 (TACC3) was observed independent of the tumor type (five squamous cell carcinomas and two adenocarcinomas) [23,24]. 

FGFR fusions were reported for the first time in 2012, in a glioblastoma tumor; they were rarely observed (0.2%) in non-small cell lung cancer, and predominantly (but not exclusively) in squamous cell carcinomas (3% of them harboring FGFR3-TACC3 rearrangement). This is an extremely important observation, since these patients could benefit from tailored therapy [23,25,26,27,28,29].

Molecular pathways associated with acantholytic, pseudoangiosarcomatous, signet ring cell, and clear cell are incompletely elucidated; at present, the only proven connections were between signet ring cell morphology-ALK rearrangement and clear cell morphology–FGFR3-TACC3 fusion.

It is very important to further analyze the lung tumors sharing these atypical features, since they may be associated with particular epidemiologic, prognostic, and genetic mutations. The most advanced studies focused on the signet ring cell morphology, which was frequently reported in lung adenocarcinomas with ALK-rearrangement, predominantly diagnosed as advanced cancer in young, never-smoker females. These patients benefit from targeted therapy with ALK inhibitors, which was associated with better survival rates and mild side effects compared to chemotherapy. Although there are many ALK inhibitors approved by the U.S. Food and Food and Drug Administration (FDA), there are many clinical trials underway to develop new medications due to drug-resistance development.

Due to significant progress in lung cancer research, oncological therapy is continuously evolving; however, there are still many aspects that need to be clarified, as our study reveals.

The study of these tumors is complex, due to their low incidence. The discovery of other genetic abnormalities with potential targeted therapies may significantly improve the overall survival and the free-disease survival of these patients, an aspect that is very important given the poor prognosis of the tumors sharing these characteristics.

The differential diagnosis of carcinomas having these atypical features may be extremely challenging, as they may represent primary or secondary lung malignancies with various origins (such as gastric, kidney, ovary, prostatic, skin, endometrial etc.,) and histogenesis (carcinomas, sarcomas, carcinosarcomas or other, even rarer, forms of cancer) [2,3,4,5,30]. As these aspects are rarely encountered in lung primary cancers, they may not have a major impact on traditional diagnostic criteria, but they may have the potential to be introduced in future WHO classifications as rare subtypes of lung NSCLC. Recognition of pulmonary origin is extremely important, as this crucially impacts the staging system (pTNM), as these tumors are frequently considered metastatic (pM1). The immunostains are very useful in confirming the pulmonary origin and the histopathological subtype of cancer, but there are no specific immunohistochemical markers or molecular tests that pathologists can use to diagnose these tumors. 

Genetic tests are important, not only for the diagnosis, but also for the treatment of these malignancies, as studies are proving an association of some rare features with particular genetic abnormalities (such as signet ring cell contingent associated with ALK-rearrangement). Considering this, it may be a potential improvement to introduce the signet ring cell and clear cell as distinct subtypes of lung non-small carcinomas. The other two patterns (acantholytic and pseudoangiomatous) have not been associated with any genetic abnormalities; thus we consider that more studies are needed to sustain their introduction as separate entities in the WHO classification.

The literature review reported a reduced number of cases sharing the mentioned atypical features. The major problem focuses on differentiating the acantholytic and pseudoangiosarcomatous carcinomas, with some authors considering these to be a unique pathology. However, we consider it important that these be reported as different entities, so that they can be separately studied to detect epidemiologic, prognostic, and genetic particularities. The pseudoangiosarcomatous pattern is the rarest, with only a few cases reported. We noticed that they share common epidemiological and clinical features: middle-aged or older patients, long-term smokers, presenting with hemoptysis, cough, pain, or weight loss. The gender ratio for the cases reported was M:F = 8:1 and the median age at diagnosis was 62.55 years old. Our patient was a female, 52 years old, who complained of hemoptysis, cough, inappetence, and physical asthenia. We did not find relevant information regarding the genetic abnormalities that could sustain these as two different pathologies, but more studies are needed. 

## 5. Conclusions

The purpose of this study was to draw the pathologist’s attention to rare and unusual microscopic aspects of some of the most common types of lung cancer, to avoid misdiagnoses, and to ensure the correct classification of the tumors. 

To our knowledge, this is the first case of lung adenocarcinoma to have been described as having a pseudoangiosarcomatous pattern. The particularity of association with Aspergillus spp. infection may influence the histopathological aspects, but we did not find solid studies supporting this. 

It is also extremely important to recognize these histopathological features, given the fact that most of these tumors have been associated with a worse clinical evolution and outcome. Two of the patients presented in our study died shortly after the diagnosis. One was diagnosed with lung squamous cell carcinoma with acantholytic features and the other with adenocarcinoma having a signet ring cell morphology; both of these were metastatic. This confirms the high rate of metastasis and the poor prognosis of these tumors.

Our study analyzed the dynamics of the WHO classification of lung tumors and the opportunity for each of these aspects to represent a particular histopathological type of lung squamous cell carcinoma and/or adenocarcinoma, as is stated currently for other organs (such as skin). We consider it very important that pathologists be aware of the rare, atypical aspects that primary lung non-small carcinomas may have. For example, even if the presence of a signet ring cell contingent strongly suggests an adenocarcinoma, this should not exclude the presence of other types of carcinomas or non-epithelial tumors. In conclusion, it should be mandatory for any tumor with an atypical morphology for primary lung cancer to be immunophenotyped. This will improve proper classification, according to the WHO, and also help to avoid misdiagnoses as metastatic tumors. In this manner, oncologists will be able to choose a correct and targeted treatment.

Even though some of the described entities share common features, in our opinion, acantholytic, pseudoangiosarcomatous, signet ring cell, and clear cell features should be postulated as different entities, as they may have different clinical and molecular behaviors. For example, some studies suggest that the signet ring morphology of the adenocarcinoma is associated with ALK rearrangement, which benefits from tailored therapy with ALK-tyrosine kinase inhibitors (ALK- TKI). Some studies suggest that clear cell morphology is associated with FGFR3-TACC3 fusion, with patients therefore being eligible for FGFR inhibitors.

In our opinion, reporting of these cases should be encouraged, because, due to the rarity of these tumoral phenotypes, additional studies are needed to better understand the histogenesis, evolution, and molecular alterations, which may in turn have important therapeutic implications.

## Figures and Tables

**Table 1:** Rare histopathological features of lung carcinoma—literature review (2013-present).

No.	Rare Histopathological Features of Lung Carcinoma	Keywords	Total Number of Articles	Number of Relevant Articles
1	Acantholytic	acantholytic, carcinoma, lung, pulmonary	8	5
2	Pseudoangiosarcomatous	pseudoangiosarcomatous, lung, pulmonary, carcinoma	2	1
3	Signet ring cell	signet ring, lung, pulmonary, carcinoma, squamous cell carcinoma, adenocarcinoma	11	5
4	Clear cell	clear features, lung, pulmonary, carcinoma, squamous cell carcinoma, adenocarcinoma	38	2

## Data Availability

Not applicable.
